# Recent Progress in Cell Therapy in Solid Organ Transplantation

**Published:** 2017-08-01

**Authors:** R. Garakani, R. F. Saidi

**Affiliations:** 1 Division of Organ Transplantation, Department of Surgery, Rhode Island Hospital, Alpert Medical School of Brown University, Providence RI, USA; 2 Department of Surgery, Digestive Disease Research Institute, Shariati Hospital, Tehran, Iran

**Keywords:** Stem cells, Organ transplantation, T-Lymphocytes, Regulatory, Immunosuppression, Immune tolerance

## Abstract

There has been ample of preclinical and animal studies showing efficacy and safety of using various cells, such as stem cells or T regulatory cells, after transplantation for tissue repair, immunosuppression or tolerance induction. However, there has been a significant progress recently using cell therapy in solid organ transplantation in small clinical trials. Recent results have been promising and using cell therapy in solid organ transplantation seems feasible and safe. However, there are more hurdles to overcome such as dose and timing of the infusions. Current studies mainly focused on live donor kidney transplantation. Expansion of current regimes to other organs and deceased donor transplantation would be crucial.

## INTRODUCTION

Solid organ transplantation is the treatment of choice for patients suffering from end-stage organ disease. However, chronic immunosuppression imposes substantial risks of morbidity and mortality, including nephrotoxicity and an increased risk of cardiovascular diseases and diabetes. Moreover, these drugs have failed to substantially prolong long-term graft survival in the past two decades, despite a dramatic improvement in short-term graft survival. The attention of transplant community has turned to find out new strategies to achieve allograft tolerance and avoid the need for long-term immunosuppression [1].

The greatest challenge facing the field of organ transplantation today is to increase the number of organs available for transplant. A variety of approaches have been implemented to expand organ donor pool including increased live donation, a national effort to expand deceased donor donation, split organ donation, paired donor exchange, national sharing models and greater utilization of expanded criteria donors (ECD) [2-4]. Although donation after brain death (DBD) accounts for the majority of deceased organ donors, in the recent years, there has been a growing interest in donors who have severe and irreversible brain injuries but do not meet the criteria for brain death. If the physician and family agree that the patient has no chance of recovery to a meaningful life, life support can be discontinued and the patient can be allowed to progress to circulatory arrest and then still donate organs (donation after cardiac death [DCD]) [5, 6].

These changes have led to increased use of marginal organs that can lead to poor outcomes and increase in resource utilization and cost [7].

Cell-based therapies have been proposed as innovative approaches to repair marginal organs, minimizing ischemia reperfusion injury (IRI) and induce immune tolerance in solid organ transplantation. The hope is that administration of cells with immunoregulatory properties to transplant recipients could tip the balance between effector and regulatory pathways, ultimately promoting the potential of the host immune system to control the immune response to the allograft. In particular, bone marrow cells, hematopoietic stem cells (HSC), mesenchymal stem cells (MSC), and T regulatory cells (Treg) are emerging as a promising cell therapy in clinical transplantation. In this article we provide an overview of clinical trials data that support the potential effects of these cells in initial clinical studies.

Clinical Studies Using Bone Marrow or Hematopoietic Stem Cells in Transplant Patients

Beginning with an observation made in mice and non-human primates, that mixed chimerism and tolerance could be induced following a conditioning regimen and bone marrow transplantation, clinically applicable regimens to induce tolerance in both HLA-matched and HLA-mismatched kidney transplant recipients have been developed at several centers [8-10].

The Stanford group has focused on total lymphocyte irradiation (TLI)-based regimen to induce tolerance. Their conditioning protocol consists of TLI and rabbit anti-thymocyte globulin (ATG) followed by infusion of CD34^+^-enriched donor peripheral blood stem cells. The patients were then started on calcineurin inhibitor (CNI)/mycophenolate mofetil (MMF)/steroids therapy until weaning was attempted several months later. They only included HLA-matched patients. Chimerism was initially induced in 21 of the 22 patients studied, and 18 met the immunosuppression withdrawal criteria. 

Their recent third cohort included 10 recipients of HLA haplotype-matched kidney on another conditioning regimen consisted of an escalating dose of infused CD34^+^ and CD3^+^ T cells to promote persistent mixed chimerism. Persistent chimerism for at least 12 months was achieved in two patients. Patients developed more than 60% chimerism that was achieved at least for 2 months in the two recipients [11-13].

A group at Northwestern University tried to induce tolerance in HLA-mismatched kidney transplant recipients using fludarabine, cyclophosphamide, and total body irradiation. This is followed by kidney transplantation, then donor HSCs. Immunosuppression consists of MMF and tacrolimus. In addition, the conditioning regimen includes infusion of a unique “facilitating cell” (a mixture of CD8^+^/TCR^-^) in an effort to enhance engraftment and further reduce the risk of graft *vs*. host disease (GVHD). Fifteen recipients were initially enrolled. Nine patients developed durable chimerism; immunosuppression was successfully discontinued in six patients [14-16].

This Northwestern group is also pursuing another tolerance induction strategy for HLA-matched kidney transplant recipients. This regimen includes alemtuzumab, donor HSCs, tacrolimus, and MMF. Tacrolimus was converted to sirolimus after three months with attempted complete drug withdrawal by 24 months. Of the reported 10 recipients, five had successful immunosuppression withdrawal for 16–36 months, two had disease recurrence requiring ongoing immunosuppression, and three had subclinical rejection on protocol biopsies requiring reinstitution of immunosuppression. None of these recipients maintained durable chimerism beyond one year [17].

The initial conditioning regimen for HLA-mismatched kidney transplantation at Massachusetts General Hospital using a non-myeloablative preparative regimen included cyclophosphamide, thymic irradiation, anti-CD2 mAb, and post-transplant CNI administration. They first studied five patients, who had end-stage renal disease, received combined bone marrow and kidney transplants from HLA single-haplotype mismatched living related donors. One patient had an irreversible humoral rejection while all patients had transient chimerism. Of the 10 recipients of HLA-mismatched kidneys enrolled in the protocol, all developed transient mixed chimerism, and immunosuppression was successfully discontinued in seven of them by 14 months after transplant. After a period of 6–13 years, four remained immunosuppression-free, whereas three resumed immunosuppression at 5, 7, and 8 years after kidney transplantation as a result of original kidney disease recurrence or chronic rejection.

Their T cells showed donor-specific unresponsiveness and there were high levels of P3 (FOXP3) messenger RNA (mRNA) [18, 19]. Table 1 summarized the results of these trials.

**Table 1:** Clinical trials using bone marrow cells in solid organ transplantation

Center	Conditioning	HLA	Cells	Chimerism
Stanford [11-13]	TLI, ATG	Match	HSC	Durable
Northwestern [14-17]	Fludarabine, cyclophosphamide, TBI	Mismatch	HSC, facilitating cell	Durable
MGH [18, 19]	Anti-CD2, TI, cyclophosphamide	Mismatch	Donor bone marrow	Transient

TI: thymic irradiation; TBI: total body irradiation; TLI: total lymphoid irradiation; ATG: anti-thymocyte globulin; HSC: hematopoietic stem cell

Clinical Studies Using Mesenchymal Stem Cells in Transplant Patients

MSC comprise a heterogeneous cell population of putative pericytic origin. The 2006 guidelines of the International Society for Cellular Therapy (ISCT) identify MSC based on (i) adherence to plastic; (ii) ≥95% of the MSC population must express CD105, CD73 and CD90; must lack expression ≤2% positive of CD45, CD34, CD14, or CD11b, CD79a, or CD19 and HLA class II; and (iii) multipotent differentiation (osteoblast, adipocyte, and chondroblast under standard *in vitro* differentiating conditions). *Bona fide* MSCs are obtained from bone marrow (BM), adipose tissue (AT), umbilical cord (UC), and other human tissues, likely due to their perivascular (pericyte) origin [20].

After inoculum, MSCs preferentially home at the site of vascular damage or inflammation where they likely function as the native resident pericytes/MSCs do in small, minor injuries. This property may help mitigating IRI, rescuing marginal donor organs, reducing activation of innate immunity leading to progressive tissue fibrosis, and blunting “danger signals” that could synergize with immune tolerance-inducing strategies. Immunomodulatory effects of MSCs have been recognized on T, B, natural killer (NK), dendritic (DC), and monocyte cell functions, as well as on the induction of “regulatory” immune circuits [20]. 

Tan and his colleagues at Fuzhou General Hospital in China, studied the possibility of autologous mesenchymal stem cells serving as a replacement of antibody induction for patients with end-stage renal disease [21]. Both at kidney reperfusion and two weeks post-kidney reperfusion, patients were inoculated with marrow-derived autologous mesenchymal stem cells (1–2 × 10^6^/kg). Fifty-three patients received standard dose and 52 patients received low-dose CNIs. The 51 patients in the control group received anti-IL-2 receptor antibody and standard dose CNIs. After six months, 7.5% of the autologous mesenchymal stem cells and standard dose CNI group and 7.7% of the low dose group had biopsy-confirmed acute rejection, while 21.5% of the control group had this type of rejection; 7.8% of patients in the control group had glucocorticoid-resistant rejection, while none of the patients in the other two groups had this type of rejection. In both MSC groups, renal function recovered faster. This showed an increase in eGFR levels in the first month post-surgery than in the control group. The results of this study indicate that MSCs rather than anti-IL-2 receptor antibody induction therapy produced a lower incidence of acute rejection, lowered the risk of opportunistic infection, and after one year, improved renal function.

A study regarding the effects of mesenchymal stromal cells in allograft rejection and fibrosis was conducted at Leiden University Medical Center in the Netherlands [22]. Six patients received autologous bone marrow mesenchymal stromal cell infusions. Two recipients had allograft rejection and received surveillance biopsies. Maintenance immunosuppression remained unaltered, while both patients had a resolution of tubulitis without interstitial fibrosis or tubular atrophy (IF/TA). Three of the six patients had an opportunistic viral infection and five of them showed a donor-specific downregulation of the peripheral blood mononuclear cell proliferation assay. The authors concluded that in transplant recipients with subclinical rejection and IF/TA, autologous bone marrow mesenchymal stem cell treatment is feasible and beneficial. 

In a study conducted at the Center for Stem Cell Biology and Tissue Engineering at SunYat-sen University [23], the use of MSC with its immunosuppressive function was studied. Donor-derived bone marrow MSCs along with a dose of tacrolimus was administered to six kidney transplant recipients. Six other patients serving as the control, received a dose of tacrolimus. Within 12 months post-kidney transplantation, the safety of MSC infusion, acute rejection, graft function, and patient and graft survival were observed. There was no immediate or long-term toxic side effects linked with the MSCs. The tacrolimus dose was significantly reduced in In MSC recipients compared with that in the control group. At the third month, patients in the MSC group had notably higher B cell levels than the control group. Furthermore, at the third month all of the patients had no chimerisms and at month twelve, all had stable renal function. The control group had one acute rejection. As a result, MSCs could reduce the dosage of conventional immunosuppressive drug in renal transplantation.

Perico, *et al* [24], assessed clinical application of MSC in transplantation. Two recipients of kidneys from living-related donors were given T cell-depleting induction therapy and maintenance immunosuppression with cyclosporine and MMF. MSCs were administered intravenously seven days after transplantation. This study also shows that MSC infusion allows enlargement of Treg in the peripheral blood and controls memory CD8^+^ T cell function. 

Vanikar studied living-donor renal transplantation (LDRT) using pretransplant stem cell transplantation (SCT) where minimization of immunosuppression was achieved [25]. Of the 916 patients who underwent LDRT, 606 patients (the test group) were under tolerance induction protocol (TIP) and 310 under triple immunosuppression of CNI, MMF, and prednisone (controls). The four-year patient survival ranged from a minimum of 82.7% to a maximum of 93.5%. The mean serum creatinine at four years ranged from 1.26 to 2.1 mg/dL. With these results, it can be said that stem cell transplantation is effective in IS minimization in LDRT. 

Perico and colleagues [26], assessed the clinical application of mesenchymal stromal cells for immunomodulation therapy in transplantation. Two kidney transplant recipients were examined whether pre-transplant (day 1) infusion of autologous mesenchymal stromal cells protected from the development of acute graft dysfunction previously reported in patients given mesenchymal stromal cells post-transplant or if avoiding basiliximab in the induction regimen improved the mesenchymal stromal cells-induced Treg expansion previously reported with therapy including this anti-CD25-antibody. A third patient received mesenchymal stromal cells treatment and graft function remained normal after one year. In the fourth patient, acute cellular rejection occurred two weeks after transplantation. CD4^+^ FoxP3^+^ Treg expansion was comparable in mesenchymal stromal cells-treated patients with or without basiliximab induction and pretransplant mesenchymal stromal cells did not affect kidney graft negatively. In conclusion, induction therapy without basiliximab is not advantageous on CD4^+^ FoxP3^+^ Treg expansion. 

Lee and colleagues [27], gave living adult donor kidney transplantation (LDKT) recipients MSCs derived from the donor bone marrow in order to evaluate the safety of immunological changes in relation to intra-osseous injection of MSCs into the bone marrow. At the time of transplantation, donor MSCs were injected into the bone marrow of the recipient. There were no local complications or graft failure. Three recipients had biopsy-proven acute rejections. The serum creatinine was a median of 1.23 mg/dL. Plasma level of IL-10 increased in patients with Treg induction. As a result, donor MSC injection is safe and there may be a link between the induction of inhibitory immune response and the clinical outcome in the MSC kidney transplanted patients. Table 2 shows the summary of clinical studies using MSC in transplant recipients. 

**Table 2:** Clinical translation of mesenchymal stem cells in transplantation

Author	Type of Cell Used	Type of Transplant	Number of Patients	Study Conclusion
Tan J [21]	Mesenchymal stem cells	Kidney	159	Among patients undergoing renal transplant, the use of autologous MSCs compared with anti-IL-2 receptor antibody induction therapy resulted in lower incidence of acute rejection, decreased risk of opportunistic infection, and better estimated renal function at 1 year.
Reinders ME[22]	Mesenchymal stem cells	Kidney	6	Autologous BM MSC treatment in transplant recipients with subclinical rejection and IF/TA is clinically feasible and safe, and the findings are suggestive of systemic immunosuppression.
Peng Y [23]	Mesenchymal stem cells	Kidney	12	These preliminary data suggest that the use of MSCs could provide potential benefits in renal transplantation by reducing the dosage of conventional immunosuppressive drug that is required to maintain long-term graft survival and function.
Perico N [26]	Mesenchymal Stem Cells	Kidney	2	Findings from this study in the two patients show that MSC infusion in kidney transplant recipients is feasible, allows enlargement of Treg in the peripheral blood, and controls memory CD8^+^ T cell function. Future clinical trials with MSCs to look with the greatest care for unwanted side effects is advised.
Vanikar AV [25]	Adipose tissue-derived mesenchymal and hematopoietic stem cell	Kidney	916	Stem cell transplantation is effective in IS minimization in LDRT resulting in good graft function and patient and graft survival at 4 years
Lee H [27]	Mesenchymal stem cell	Kidney	7	Donor MSC injection into the iliac bone at the time of kidney Tx was feasible and safe. A possible correlation was observed between the induction of inhibitory immune responses and the clinical outcome in the MSC-kidney transplanted patients. Further research will be performed to evaluate the efficacy of MSC injection for the induction of mixed chimerism and subsequent immune tolerance.

Clinical Studies Using T Regulatory in Transplant Patients

As a control mechanism for unrestrained effector immunity, regulatory cells are essential to maintain equilibrium of the immune response. Naturally present in the immune system, thymus-derived Treg (nTreg) conserves tolerance of self-antigens and contains responses to them. Additionally, T-cell differentiation in a peripheral tolerogenic environment produces Treg, frequently referred to as “induced” or “peripheral” Treg. Treg express CD25—the α-chain of interleukin (IL)-2 receptor—as well as the transcription factor fork-head box P3 (FoxP3). Induced Treg promotes immunologic unresponsiveness to alloantigens, a process that was initially identified in mice, and was subsequently appreciated in humans [28].

Regulatory T cells have been detected in the peripheral blood of transplant recipients, as well as in the allograft, resulting in the suggestion that they play a role in the process of allograft acceptance that lessens immunological responses of acute rejection over time. But recipients lack adequate Treg to prevent rejection at the time of transplant when effector immune cells dominate the immune response to alloantigen. Expanding the Treg population and shifting the immune balance in favor of the regulatory cells has been proposed as a strategy to potentially minimize the need for effector immunity control, reduce immunosuppression requirements, and induce tolerance.

In small animal studies, CD4^+^ CD25^+^ FoxP3^+^ Treg cells have been infused to effectively prevent acute and chronic rejection. Clinical evidence for such regulatory mechanisms is drawn from observations in recipients who discontinue their maintenance immunosuppression (due to medical necessity or noncompliance), but do not reject their allograft. The so-called “operationally tolerant,” Brouard, *et al*, observed some of these individual display T cells, both CD4^+^ and CD8^+^, with regulatory properties. We have also studied regulatory signals in our tolerant cohort, and noted an increase in intragraft FoxP3, supporting the hypothesis that peripheral regulatory mechanisms play a role in the maintenance of unresponsiveness.

Clinical application of Treg to ameliorate GVHD in bone marrow transplantation has been reported recently, there has been significant progress made clarifying the conditions required for expansion of nTreg from the resting population of 1.3 × 10^10^/body to more than 5–8 × 10^10^/body. The hypothesis is that this dose of Treg could effectively harness effector T cells, if infused *in vivo*. As a result of this technological progress, a multinational clinical study has been designed. The One Study involves eight clinical centers in Europe and the United States. In this clinical trial, an identical basic immunosuppression protocol for HLA-mismatched kidney transplantation will be used in all centers, and the effect of addition of nTreg to randomly selected recipients has been evaluated [29].

In conclusion, there has been a significant progress using cell therapy in solid organ transplantation from preclinical phase to a clinical reality. Recent results have been promising and using cell therapy in solid organ transplantation seems feasible and safe. However, there are more hurdles to overcome such as dose and timing of the infusions. Current studies mainly focused on liver donor kidney transplantation. Expansion of current regimes to other organs and deceased donor transplantation would be crucial.
